# Rationality versus reality: the challenges of evidence-based decision making for health policy makers

**DOI:** 10.1186/1748-5908-5-39

**Published:** 2010-05-26

**Authors:** Deirdre McCaughey, Nealia S Bruning

**Affiliations:** 1 Department of Health Policy and Administration, The Pennsylvania State University, State College, Pennsylvania, USA; 2 I.H. Asper School of Business, University of Manitoba, Winnipeg, Manitoba, Canada

## Abstract

**Background:**

Current healthcare systems have extended the evidence-based medicine (EBM) approach to health policy and delivery decisions, such as access-to-care, healthcare funding and health program continuance, through attempts to integrate valid and reliable evidence into the decision making process. These policy decisions have major impacts on society and have high personal and financial costs associated with those decisions. Decision models such as these function under a shared assumption of rational choice and utility maximization in the decision-making process.

**Discussion:**

We contend that health policy decision makers are generally unable to attain the basic goals of evidence-based decision making (EBDM) and evidence-based policy making (EBPM) because humans make decisions with their naturally limited, faulty, and biased decision-making processes. A cognitive information processing framework is presented to support this argument, and subtle cognitive processing mechanisms are introduced to support the focal thesis: health policy makers' decisions are influenced by the subjective manner in which they individually process decision-relevant information rather than on the objective merits of the evidence alone. As such, subsequent health policy decisions do not necessarily achieve the goals of evidence-based policy making, such as maximizing health outcomes for society based on valid and reliable research evidence.

**Summary:**

In this era of increasing adoption of evidence-based healthcare models, the rational choice, utility maximizing assumptions in EBDM and EBPM, must be critically evaluated to ensure effective and high-quality health policy decisions. The cognitive information processing framework presented here will aid health policy decision makers by identifying how their decisions might be subtly influenced by non-rational factors. In this paper, we identify some of the biases and potential intervention points and provide some initial suggestions about how the EBDM/EBPM process can be improved.

## Background

High expenditures in healthcare have stimulated healthcare policy makers to explore more effective and efficient healthcare delivery options. For example, in 2008 national health expenditures in the US were $2.3 trillion, or $7,681 per person on average, and accounted for 16.2 percent of the gross domestic product (GDP) [1]. This figure is expected to reach 19.3 percent of GDP by 2019, or approximately $4.5 trillion, the highest per capita expenditures in the world [1]. Given the high societal costs of healthcare and potential benefits of improved delivery and enhanced population health, strong incentives exist to improve health policy decision making. In the global health arena, numerous individual, political, and market forces influence the traditional health policy decision making environment [1-5]. While many forces influence policy making, this article focuses on the influence of individual cognitive information processing. Research investigating individual decision making has identified cognitive information processing as a key factor in the decision-making process [6-8]. A cognitive information-processing approach accounts for internally generated mechanisms by which relevant decision-making information is processed by individuals and individuals participating in group decision making [9,10]. This is in contrast to externally generated mechanisms of influence, such as political will, interest groups, and economic factors [3-5].

Understanding a health policy decision-making task requires policy makers to recognize various individual factors that influence their decision making, both individually and when in groups [11-13]. As such, public health policy is a valuable context in which to consider the role of cognitive processing of decision information. While competing influences on decision making are not new topics, the recent emphasis in public policy on evidence-based decision making (EBDM) and evidence-based policy making (EBPM) reinforces the need to examine some of the factors that bias the decision-making process. We believe recognition of the mechanics of cognitive processing will assist health policy makers in identifying how their policy decisions are internally influenced, and how decisions might be subsequently improved.

**Figure 1:**
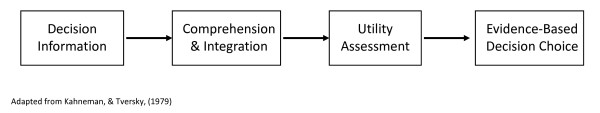
**Evidence-Based Rational Choice Decision Model**.

In many countries, the nature of public policy dictates that health policy makers are subject to decision influences from different stakeholders, including the media, public opinion polls, funding agencies, managed-care organizations, and special interest groups [4,5,13-20]. In addition to various stakeholders, policy decisions are subject to judicial rulings, political mandates, policy legacies, perceptions of policy importance, and, most currently, the growing drive to utilize an evidence-based approach to health policy making [3,13,21-27]. These myriad of influence sources can be classified as external information that policy makers must cognitively process in order to arrive at a final decision. In addition, many models guiding the policy making process assume policy makers are capable of accurately analyzing decision information, understanding the relevant evidence, are resistant to influences and biases, and seek to make decisions that maximize societal benefit [5,19,27,28]. These assumptions are essentially the hallmarks of linear, rational policy objectives, mirror the dynamics of rational choice decision models (Figure 1), and also reflect many of the tenets of EBDM and EBPM [2,5,13,14,24-27]. However, these objectives and models collectively fail to consider the decision-making literature, which shows these assumptions are problematic, incomplete, and, in some cases, false [19,29-33].

Utilizing health policy decision making as a basis, this article presents a theoretical decision-processing framework that supports the focal thesis: during the health policy process, decision makers are subjectively influenced by the manner in which they cognitively process information. Articulating cognitive processing barriers that policy makers experience in real-world decision choices and in the context of the rigorous demands of evidence-based decision and evidence-based policy making (hereafter referred to as EBDM) will challenge many of the assumptions that health policy making is strongly guided by research [13,15,22,23,34,35]. Recognizing and understanding cognitive processing limitations and biases may facilitate a more realistic evidence-based approach in all facets of health policy decision making [5,22,24,25,36-38].

## Discussion

### EBDM: The challenges of rational choice

Numerous healthcare systems exist globally, yet many of the same factors influence the direction of health policy regardless of national boundaries. Factors include diversity in healthcare coverage, societal demands for the provision of healthcare, technological advances in diagnostics, quality of care initiatives, and a rapidly changing healthcare workforce [2,4,13,18,39]. Some argue that one of the strongest forces driving health policy change is the dissemination and adoption of evidence-based medicine (EBM) and EBDM practices within health systems [3,16,25,38,40]. The growing prominence of EBDM in healthcare and health policy is due to such factors as cost considerations, the increasing prevalence of managed care organizations and third party payers, the need to ensure appropriate usage of health interventions, and public calls for accountability and affordability [13,18,25,40]. Public policy literature has indentified that numerous key decision makers believe evidence-based health policy and the inclusion of evidence in public policy making is both a desirable and an attainable policy goal [13,16,25].

While EBDM offers potential value in enhancing public policy, by its nature it assumes a degree of individual rationality in the decision process on the part of decision makers [16,24,41,42]. However, decision-making research has shown that relevant data may be distorted and/or ignored while decision processing is occurring [24,42-44]. Given that EBDM is increasingly called for in key health policy decisions, such as resource allocation, program determination, funding, and measuring program effectiveness[14-16], it is critically important to examine the mechanics of information processing and decision making in order to guide successful EBDM [18,24,43].

The rational choice principle that governs EBDM assumes that policy makers have the required cognitive abilities and knowledge to interpret, process, understand, and determine the validity of scientific evidence relevant to policy decisions [2,16,33,45]. However, decision-making research has shown that decision makers, even if they have access to required information and have relevant expertise, may not engage in complex cognitive information processing when making decisions [13,15,44,46-50]. For example, cognitive processing research has identified both bounded rationality and 'satisficing' as limitations to complex cognitive processing [2,15,44,46-50]. Bounded rationality defines the situation where decision makers are limited in their abilities to search for a solution; therefore, they 'satisfice', by choosing the first alternative that meets or 'satisfies' minimum criteria for solving the problem rather than continuing the search for the optimal solution [2,13,32,44,46,49,50]. Satisficing alternatives may be subject to a number of diverse influences, which support the position that policy makers can be subject to non-rational decision influences [13,25,41,47,51-53].

The nature of cognitive information processing is further highlighted in one stream of the public policy literature that argues that relevant research is frequently ignored by policy makers [15,25,29,38,40,53]. The plethora of evidence and the variety of methods by which evidence is presented (*e.g*., randomized clinical trials, systematic reviews, and qualitative case studies) compounds the uncertainty for policy makers in attempting to assess 'what is evidence' and how to assess the strength of the evidence [13]. For example, one critical factor that has arisen is the question of the policy makers' ability to judge the quality and applicability of research results [13,16,25,38,40]. Issues such as study results emanating from multiple scientific disciplines, use of specialized jargon, and sophisticated statistical analyses can impede policy makers' understanding [13]. As such, it is posited that numerous individuals do not have the broad ranging expertise to adequately assess scientific information across health policy domains, thus they will satisfice their decision information needs and rely on secondary sources that summarize research results and translate the findings into 'lay' language. In other words, the assumed rational, utility maximizing decision-making processes begin to break down.

With respect to the value or utility of a decision, the nature of democratic political systems endorses policy makers' efforts to pursue maximal public satisfaction with government decision making [4,16,30,54-56]. Utility maximization originates in expected utility theory, which contends that a decision maker will make a rational choice to maximize his/her utility (gain) by choosing the decision option with the greatest probable gain [47]. If public policy models imply that policy makers seek to attain greatest societal utility, another assumption is being made regarding the rationality of public policy decision making [25,30,54,57]. Decision-making research has demonstrated that a decision maker's utility is highly subjective and may include variables, such as personal gain, risk tolerance, relevance to related events, and value of a decision to the organization [22,28,44,46,47,54].

Complicating the picture further is the observation that policy makers are forming policy in response to and in conjunction with groups of individuals, all with individual objectives and biases. Group decisions are argued to be superior to individual decision making in that they tap into a wider knowledge base, generally create more information, and theoretically are more open to decision information examination [58,59]. However, there have been many studies demonstrating group decision phenomena, such as groupthink and non-rational escalation of commitment, which exhibit cognitive decision-making behaviors that impede and prevent rational decision choices by groups [58-60]. While the nature of decision making in groups is outside the focus of this paper, it is key to note that groups are comprised of individuals. Therefore, despite the expectation of rationality in policy decision making, policy makers' decisions can include individual and group utility factors and be a source of bias because decision information is rooted in individual cognitive processing [44-50,61].

In summary, health policy makers are charged with the responsibility of making effective and utility maximizing policy decisions regarding their respective health systems in a theoretically evidence-based environment [3,13,20,40]. Yet, many authors argue that the nature of the milieu in which healthcare decisions are made, the limited understanding of the decision makers regarding their own biases, and the complexity of evidence does not support a direct translation of research evidence into decisions [13,19,41]. Therefore, despite the positive intent of EBDM, health policy outcomes may actually be, to a varying extent, subjectively derived [22,23,33,40,45,61]. We argue that the use of research in policy decision making should not focus on whether evidence is used but how evidence is processed to inform decision making and the contexts in which decision making occurs [3,23,61]. In order to meet health policy objectives such as evidence-informed or evidence-based decisions, there must be a clear understanding of how individual cognitive processing influences the decision-making process [62]. Given the extremely high and increasing costs of healthcare, we hope that improvements in the health policy decision-making processes will yield positive returns to society and its citizenry.

### Cognitive information processing framework

**Figure 2:**
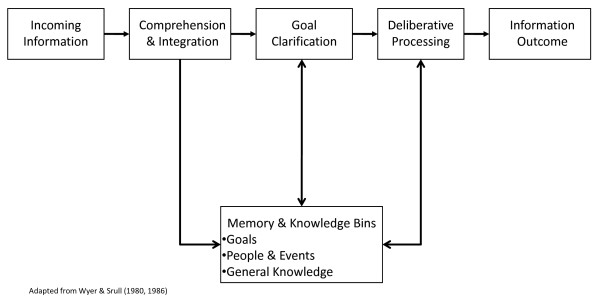
**Cognitive Processing Model (Deliberative Stage Only)**.

Social information processing models view cognitive processing as occurring in two stages [9,10,63-65]. Wyer and Srull [10] have proposed one of most recognized information processing models, which will be used here to provide the structure for the basic cognitive information processing discussion (Figure 2). The first stage, entitled the 'spontaneous stage' (a non-processing, automatic function) will be briefly discussed here. Intervention at the automatic stage is more challenging because the stage involves almost reflexive perceptual mechanisms. The second stage, entitled the 'deliberate stage', involves more active information processing. During this active processing, individual biases and subjectivity can be identified as information processing drivers known to influence decision making and, thus, will be the focus of this paper.

In Wyer and Srull's [10] deliberate stage of information processing, the major purpose is to articulate how individuals pursue their goals and objectives (may be conscious or subconscious) through the manner in which information is processed. Goals can be general (*e.g*., form an impression about an event/person), or they can be quite specific (*e.g*. decide what course of action to take to resolve a problem). The cognitive interaction between goal identification/clarification and deliberative processing is such that the information subsequently recalled and the resulting decision is directly reflective of the information processing objectives [9]. For example, the objective to evaluate whether a health policy is effective (*i.e*., has it resolved the identified health problem) may lead policy makers to pay attention to different aspects of the policy information and process the information differently than if the objective is to determine whether the policy fulfills the election mandates of the governing party.

In other words, incoming raw information in the automatic processing stage is interpreted, categorized, and encoded. Information requiring no further processing and having no link to a current goal requiring further deliberation generates an automatic response and exits the cognitive processing cycle [9,63]. However, information identified as relevant to an existing objective or goal proceeds to the deliberative stage, or 'cognitive working space' [10]. At this stage, goals drive the cognitive search for memory and knowledge with which to process incoming information [63]. The nature of goals as drivers of information processing suggests that goals filter information processing and determine what information is attained, retained, and utilized. The attachment of individual goals to the processing of information presents an opportunity for subtle influence on policy decisions. For example, how individuals define policy goals such as those with a 'greatest societal benefit' maxim will influence how information is further processed.

According to the Wyer and Srull model [10], once in the deliberative processing stage, information that requires greater conceptualization and sense making is compared to existing categories in memory, called storage bins. These memory or storage bins contain categories of individual knowledge, including general knowledge, goal knowledge, and person/group/event knowledge. Retrieval of information from memory bins is thought to be triggered by new information that matches existing representations of previous experiences and information [9,10]. Included in the storage bins are schema, which associate different pieces of information together. For example, health policy makers seeking to make policy determinations regarding healthcare for children may have existing knowledge of policies relevant to that population group in memory storage that is then brought forward as matching information. General knowledge contains one's information about how the world functions. Goal knowledge consists of information one possesses about typical goals individuals have in specific circumstances and the means by which these goals influence information retrieval and evaluation. Information is processed to support the attainment of relevant goals. Person, event, and group knowledge, commonly organized as schema, consists of knowledge about typical representations of the specific person, event, or group. In the health policy maker example above, in a 'children' schema, decision makers may have stored information about generalized characteristics of the children group that might affect their policy decision-making process. (For a more complete discussion of social information processing and memory bins, please see Wyer and Srull, 1986). Memory bins act as a source of personal experience and knowledge and tend to guide decision making in healthcare environments [40].

The comparative process that links new information with existing cognitive representations (*e.g*., schema) captures the concept of cognitive testing for information validity. Cognitive representations are drawn from memory and matched with new information. Judgments about similarity to representations of existing knowledge (general, goal, person/group/event) might lead to comprehension and, more importantly, validates the new incoming information [9,10]. Ultimately, deliberate processing results in a final cognitive outcome that allows a decision maker to reach a conclusion, impression, or decision that is directly related to his/her previous experiences and biases. Thus, the decision-making process is substantially more complex than suggested by assumptions governing evidence-based rational choice decision models. Moreover, the very nature of cognitive processing highlights the role of internally generated influences that occur during cognitive processing, influencing a policy maker and serving as a source of non-rational decision making [28,32,46,47].

### Cognitively generated decision-making influences

**Figure 3:**
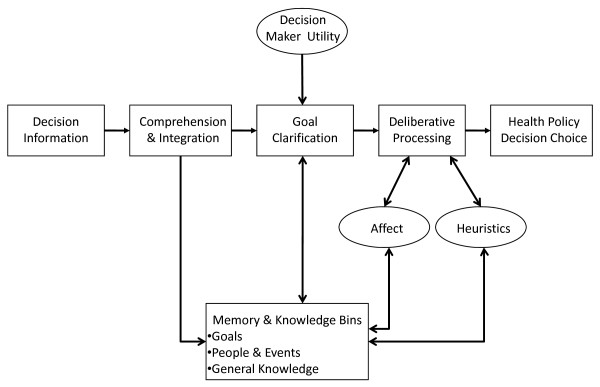
**The Cognitive Information Processing Framework for Health Policy Decision Making**.

Research into cognitive processing has identified three major sources of influence on how information is processed and evaluated: decision maker utility, affect, and heuristics [66-69]. The following sections articulate how these factors function within a cognitive information processing model (Figure 3), and how they influence the identification and evaluation of decision evidence in ways that may subtly influence health policy decision making.

### Decision maker utility

Many policy theorists call for policy making to focus more on understanding the decision process rather than on making decisions that seek maximization of societal utility [30,31,54]. We would argue that understanding and improving the decision making process and clarifying policy goals could help generate policies more attuned to both societal and individual needs. Furthermore, the decision-making literature has identified that the utility of a situation to a decision maker can ultimately influence his/her decisions [6]. Personal utility influences internally generated mechanisms in the policy decision process and is described as the individual's subjective utility.

Expected utility theory posits that decision makers facing decision alternatives will evaluate each alternative independently, with respect to perceived value and the probability of occurrence. These 'computations' result in a final value attached to each option that identifies a maximal gain choice [47,70-72]. Prospect theory, however, demonstrates that a decision maker's perceived utility can be subjectively influenced by the manner in which the information is framed (as a loss/gain or risk/no risk), what reference is being used to evaluate the options, and the relationship/salience of the alternative to the decision maker [47,70-74]. Prospect theory argues that a decision maker's utility derives from different cognitive evaluations of each prospect (decision option) and is reflective of how the options are framed (for a detailed account of the cognitive processing and prospect evaluation, see Kahneman and Tversky, 1979). Decision-making research has demonstrated that individual utility is a subjective factor and is influenced by personal preferences, desires/wants of the decision maker, degree of emotion involved in the decision, the degree of decision risk with respect to outcome certainty, and personal values [46,48,70,75].

The nature of decision maker utility is such that policy makers might experience differing utility perceptions when considering policy options, and thus be subject to varying, subtle influences. The classic decision-making example of these utility influences is Tversky and Kahneman's [28] Asian disease problem, which demonstrates that the manner in which a health problem is framed can elicit different responses to the same problem. In the original study and numerous replications, participants are presented with two choices of health programs to combat a theoretical disease outbreak [28,72,74]. The same problem and numeric outcomes are presented; however, one program's outcomes are presented as number of lives saved while the other program's outcomes are presented as number of fatalities. Consistently, the majority of participants will select their program choices based on how the information regarding lives saved/fatalities rates is framed [28,70,72-74]. The Asian disease example clearly demonstrates the influence of framing on decision alternative utility assessment and exemplifies how evidence is subjectively interpreted and used to make healthcare decisions. Other studies have demonstrated that manipulated information related to the perceived utility of a decision option can evoke inconsistent preferences or preferences that vary based on how the information is presented or framed. These inconsistencies have been shown in mental health policy, surgical interventions, and government regulations [32,67,70]. Furthermore, policy makers in healthcare have been found to incorporate their self-interests (their personal utility) when prioritizing and developing policy [22]. Personal utility assessments often cloud relevant societal level assessments of policy alternatives and/or drive the overall assessment of decision options. Thus, individual utility evidences the power to override the laudable public goals of maximizing societal utility when policy decision making takes place.

Following the tenets of social information processing theory and research supporting prospect theory, the nature of goal-directed cognitive processing suggests that a decision maker's utility is governed by his/her goals, which can be subjective in nature [10]. Inclusion of a subjective utility function as part of a cognitive information processing framework is necessary to more accurately understand health policy decision making. We argue that utility perceptions of decision makers are governed by goals retrieved during the goal-directed information processing stage and influence which information is retrieved and how it is evaluated. The evidence supporting utility as a subjective factor and its amenability to manipulation leads to the following proposition:

Proposition 1a: Policy decisions may be more likely to represent individual (identified by the policy maker's goals) rather than societal utility and are more likely to be supported than a policy decision presented as being a rational, societal utility-maximizing choice.

Proposition 1b: Policy decisions related to decision maker's experience (linked to individual memories stored in cognition) are more likely to be supported than those that are abstract or remote to the decision maker's experiences.

Thus, the above propositions suggest that the manner in which policy questions are framed and policy maker experience will influence decision utility assessments and subsequent choices regarding health policies.

### Affect

With respect to decision making, the influence of affect on individuals has been shown to influence the manner in which individuals perceive situations, the motivation of decision behaviors, the degree of decision risk tolerance, and the level and type of information recall people exhibit [6,76-80]. Research has identified both state and trait sources of affect [81-83]. State affect is the transient, short-term mood, while trait affect (typically referred to as positive and negative affect) is the more global overall mood that tends to be stable over time [82]. Individuals high in positive affect tend to reflect enthusiasm, alertness, and a positive outlook on life, while individuals high in negative affect tend to experience dissatisfaction and distress and have a poor outlook on life [69,73,82-84]. State influences are generally less reliable, stable, and predictable than trait influences; thus, they are more resistant to decision-making process improvements [81,83]. While much research into affect and cognition focuses on the influence of induced transitory mood (state), we focus here on the long-term effect of one's trait affect on cognitive processing due to the more stable and predictable nature of trait affect [83,84]. The focus on trait affect in behavior and cognitive processing is critical, given that affect has been shown to play a dominant role in both decision making and organizational outcomes [68,81,84-86].

Trait affect research identifies both positive and negative affect as influences on cognitive processing and decision-making behavior [69,81-84]. Affect has been found to act as an influence on perceptions of risk, event certainty, and gains/losses, thereby influencing the individual's perceptions and subsequent decision choices [68,73]. Individuals with high positive affect are more likely to perceive risky situations as being more certain and are less likely to believe that risky decisions will create negative personal outcomes than negative affect persons. Other studies measuring perceptions of an organization's strategic business environment found high negative affect individuals were more likely to have poor perceptions of the organization's performance, potential industry growth, and industry complexity [73,87,88]. Similar results with negative affect individuals have been found with perceptions of job performance and work attitudes [69,83,85]. The affect literature supports the conclusion that trait affect is a robust phenomenon that influences the decision-making process.

Social information processing models postulate that affect-related concepts are stored in permanent memory bins in much the same fashion as knowledge and experiences [10]. Affect is labeled and stored as specific representations, such as happy, angry, or sad. These emotions can be labeled in permanent memory as independent feelings or as associations with previous events and experiences. If a goal-directed information process is triggered by affect, it is highly probable that a different memory process will occur than a goal-directed process with no affect. Individual affect can then serve as a driver and/or a filter of the memory search. Affect is an important component of deliberative information processing and is likely a key influence in complex cognitive tasks such as deliberative decision making [63,88-90]. In general, positive affect has been shown to trigger quicker, more flexible, and more efficient processing strategies. Conversely, negative affect tends to trigger slower, more systematic, and more analytical processing strategies [6,77,79,88-92]. In addition, personal importance mediates the affect-cognitive processing relationship during decision making when greater personal importance encourages decision makers to utilize self-serving judgment strategies [93]. For example, individuals with high levels of negative affect are more prone to make biased choices when decisions were personally relevant [91].

While affect and policy decision making has not been extensively studied, based on the strength of the evidence supporting affect as an influence on cognitive processing, the following exploratory propositions are presented:

Proposition 2: Policy makers' trait affect will influence the degree of risk tolerance and uncertainty they will allow in supporting/devising new policies. Those high in positive affect are more likely to support policies with high risk and high uncertainty, while those high in negative affect are more likely to support policies with minimal risk and minimal uncertainty.

Given that many health policy decisions are fraught with emotional subtext, the above propositions add to our understanding of the mechanics of cognitive information processing through the recognition of individual affect as an influence in the cognitive processing/memory search process during decision making. Affect can and does serve as a subjective force on policy makers during the health policy decision process.

### Heuristics

The final area of influence included in the cognitive information processing framework is heuristics. Cognitive processing research has found that one's repetitive use of specific procedures and knowledge results in automatic ways to process information [64-66]. In complex decision situations, this automatic processing becomes a dominant force in information processing and results in cognitive shortcutting tactics. This behavior has major implications for the rationality assumptions of EBDM.

Heuristics are cognitive processes where full information processing requirements are bypassed and mental shortcutting occurs [66,71,73,94]. Heuristics are mental 'rules of thumb' that make decisions easier by reducing the complexity of information processing. They operate through the use of categorization to interpret information. New information is categorized based on familiar knowledge drawn from memory bins and results in more automatic processing than would normally be required [10]. Although there are many different heuristics, they are categorized based on the similarity of types of cognitive processing being utilized [66]. The three main categories of heuristics include availability, representativeness, and anchoring and adjustment [10,66].

The availability heuristic is the tendency for a decision maker to assess the frequency, probability, or likely cause of an event based on similar occurrences readily accessible in one's memory bins. Availability exerts a strong influence when the event evokes vivid emotions and is easily recalled [66]. Many media reports tend to exhibit a certain degree of sensationalism or priming that helps foster an availability heuristic [95]. For example, a health policy decision regarding the distribution, labeling, and storage restrictions of lethal drugs in hospitals will likely be strongly influenced if the media has recently presented a story about recent deaths that have occurred in emergency rooms from a mix-up between sodium chloride and potassium chloride. This example highlights the observation that decision makers spend considerable time and energy on a policy decision when linked to recent dramatic events profiled in the media [2,3,5]. While serious drug interactions or mix-ups are a rare occurrence, many media stories about healthcare system efficacy include a dramatic, emotional component that can easily trigger an availability heuristic in related decision situations.

The second heuristic, representativeness, occurs when decision makers' form their judgment of an event/target based on the perceived similarity of the event/target's attributes to a pre-existing prototypical category. In doing this, statistical probabilities are erroneously discounting [66]. For example, a policy maker may decide in favor of a health policy supporting mandatory immunizations for meningitis based on the successful implementation of other childhood immunization policies that have helped minimize the spread of contagious diseases among children (*e.g*., measles). The policy maker may then fail to account for the risk factors associated with contracting meningitis, which are statistically less probable than risks associated with contracting other contagious diseases such as measles [96]. Using the representativeness heuristic, the policy maker's decision is influenced by a simplistic cognitive shortcut that fails to consider relevant and potentially critical evidence.

Finally, the third heuristic, anchoring and adjustment, involves a decision maker's utilization of a personally relevant initial value (derived from memory) as an initial determination point about the value of a decision assessment [66]. Subsequent assessment of each decision option's value is adjusted based on the initial anchor point that the decision maker identified. For example, a policy maker determines amounts of financial support for a regional health authority using the previous budget to set the current financial budget irrespective of need, extenuating circumstances, or technological requirements. This results in potentially irrelevant data being used to determine the value and outcome of a key decision alternative, such as future budgeting and healthcare resource spending.

The utilization of heuristics in decision making has been shown to be a robust source of influence in the assessment and judgment of decision options, such as the likelihood of contracting a disease, identifying probabilities of accidental fatalities, information identification, and pharmaceutical risk [66,71,73,75]. Cognitive heuristics serve as a trigger to a prototypical representation of a situation/decision, thereby creating a judgment or response based on memory bin representations from previous experiences rather than a judgment based on the evidence of the current situation [9,10]. This linkage of decision-making heuristics to experiences during cognitive information processing supports the following proposition:

Proposition 3: Policy makers who are presented with cognitively difficult policy information and who have available in their memory a relevant heuristic will utilize that specific cognitive shortcut to support the presented policy, while those individuals who do not have an available relevant cognitive heuristic will be less likely to use a heuristic in support of the presented policy.

The purpose of discussing information processing is to comprehend how incoming information and cognitive shortcutting are common occurrences that simplify cognitive processing demands [9,10,32,44,48,64,73]. Given the complexity of most nations' health system challenges, cognitive shortcutting by policy makers is to be expected. However, one must be mindful that cognitive shortcuts do not ensure that the final decision best resolves a problem, and cognitive shortcutting fails to follow the expectations of EBDM [66].

## Conclusions

Evidence-based health policy can alter the manner in which healthcare policy is presently administered, and its growing prominence in many healthcare systems warrants examination. However, the policy process, irrespective of the nation or health system, is not a linear, rational model in which an idealized solution for a public problem can be ascertained and optimally implemented [13,19,30]. In this era of increasing prevalence of EBDM, the rationality assumptions in EBDM must be challenged to ensure effective policy decision making and high quality care for all citizens.

This paper has argued that cognitive information processing is fraught with many opportunities for subtle factors to influence policy makers' assessment of decision options. These factors are then likely to influence the resulting policy decision in a manner that is inconsistent with many of the evidence-processing expectations of EBDM. Given consideration of the complexity of cognitive information processing and the role of individual goals in how information is being processed, it is not surprising that health policy makers would readily adopt cognitive processes that simplify decisions. The cognitive information processing framework for health policy decision making presented here (Figure 3) depicts how health policy decisions might be subtly influenced by non-rational factors. Even when policy makers do not make decisions in isolation, individual subjectivity and potential biases enter the group decision process, thus influencing the outcomes.

The multi-billion dollar question is how can cognitive information processing be improved in order to ultimately lead to better health policy decisions? The information presented and the propositions presented highlight weaknesses in the decision-making process. Many organizations and agencies have policy enhancement strategies already in place [13], so the comments here are directed towards two overarching components of EBDM and, ideally, will aid in improving current decision-making practices. The first component, what is the nature of the evidence being created by researchers to be utilized in EDBM, and the second component, what practices can foster better decision making on the part of the policy makers:

1. Within the first component, an initial challenge arises around the manner in which health services research is conducted. As healthcare is a multi-sector industry, it draws health services researchers from a wide variety of health and social science disciplines (*e.g*., management, economics, political science, sociology, nursing). Deriving from these various epistemologies, research is theorized, conducted, analyzed, and evaluated using many different methods [97,98]. As a result, studies, methods, and subsequent findings may or may not be accepted as valid based upon one's philosophical and theoretical orientation regarding science [97,99]. This compounds the dilemma of defining evidence and identifying superior evidence to be used in EBDM [13]. Evidence, as we know, is a major element of EBM (the precursor to EBDM), and the hierarchical evidence spectrum argued by Sackett and others highlight Randomized controlled trials (RCTs) and meta-analyses as the gold standard of evidence [100]. This EBM foundation privileges positivist science and diminishes research conducted outside the empirical, quantitative perspective to being of lesser value, an unfair and unfounded position. As researchers are the individuals who produce most of the evidence, it is incumbent for these individuals to orientate themselves to the philosophy of science in order to gain an appreciation for the myriad of paradigms vis-à-vis the basic question of what is knowledge, what is science, and what is evidence [101]. The outcomes of this imperative academic exercise should see health services researchers embrace various research methods and the validity of findings across the research spectrum, thereby minimizing some of the existing confusion surrounding the question of what is good evidence and what evidence should be used.

2. Continuing within the first component, the second challenge derives directly from the first--translating research findings into evidence that is amenable to the end-users. In this call, we define the end-users of health services research to be decision makers, managers, politicians and others rather than the practitioners who utilize research for clinical practice from such sources as the Cochrane Collaboration [13]. Many researchers have highlighted the myriad of difficulties translating health services research into information readily understood and useable by the health services community [13,100,102]. As such, it becomes vital that health services researchers pursue improvements in how they prepare and report research for the end-users, including actions such as:

a. Linking research projects to end-users through needs analyses and the inclusion of end-users in the research agenda/program. This will aid in articulating the context of the research, identifying the relationship and purpose of the research to key stakeholders, and explicate how the findings can translate into meaningful policy achievements. These actions should then serve to create a mutually beneficial relationship with both parties having an investment in seeing the research findings utilized.

b. Preparing research findings for dissemination with sensitivity to language, inferences, and assumptions typically found in academic writings. Expecting end-users to have a full comprehension of 'research speak' sets up the dissemination mode for ineffective translation as certainly as would it be if health services researchers were expected to have full comprehension of the language, jargon, and acronyms commonly used in 'med speak'. The ability to ensure data, findings, and reports are expressed in commonly used language will aid decision makers to use the available evidence. Additionally, this may help alleviate situations in which decision makers are attempting to utilize evidence with conflicting information and conclusions.

3. Within the second component, fostering improved decision making, the next challenge is finding a balance between individual utility assessments and stakeholder utilities. To improve decision making, there are a number of suggestions and improvements to pursue including:

a. Given that policy making does not occur in isolation, it is important to identify the components of the network that are relevant and require consideration (*e.g*., institutions, industry, organizations, affiliates, government departments, fiscal budget constraints). Within that, coordination of information gathering and clarification of policy objectives that articulate the goals and objectives of the various stakeholders will help to define the utility objectives of a given policy. Using this information, policy direction can then be orientated to achieve the desired outcomes for the various stakeholders.

b. Assessment of the policy alternatives by stakeholder groups with diverse interests and objectives. Independent reviews will assist with critical review of government policy and help to promote policy that best meets public needs and maximizes the utility of broader stakeholder groups.

c. Policy implementation and subsequent outcomes require in-depth scrutiny and evaluation to ensure the policy is meeting its initial objectives. While 'policy evaluation' modes are often found in many policy models, the consistency of evaluation and response to such evaluations are often cursory and, many times, ineffective [13,19,25]. Involving stakeholders to become part of the policy creation process naturally leads to their participation in the evaluation process. Having this added element will help to ensure that thorough evaluation does occur, reflects the outcomes attained, and maximizes stakeholder utility.

4. Continuing within the second component (improved decision making), another challenge involves the actual decision-making process when groups are involved [13,19,25,103]. Group decision making has its own limitations (see Bazerman, 1998, for in-depth discussion) and decision processes need to be balanced with effective group decision making tools [58,104].

a. Decision-making processes within groups often involve either a process of inquiry (collaborative problem solving) or a process of advocacy (a function of persuasion and opinion influencing). Clearly identifying the nature of the policy decision will help direct the roles of the participants toward seeking ideas and solutions versus efforts to polarize the group toward one or two outcomes. Specific goals and direction must be spelled out to the involved group(s) in order to ensure the decision process, whether problem solving or persuasion, fulfills the overarching policy objectives [103].

b. Utilizing structured group decision-making processes will assist in minimizing the common traps of group decisions, such as non-rational escalation of commitment and the groupthink phenomenon [58,96,104]. For example, establishing a set time for problem identification, solutions, and discussion, utilizing actions to combat the groupthink, such as designating specific individuals to function as 'devil's advocate', encouraging dissent and debate to optimize productivity, identifying and curtailing pressure for conformity, and recognizing the political vulnerabilities with the group(s).

c. Controlling the structure of the group and the individuals who comprise the decision-making body will help ensure diversity of utility, needs, experience, knowledge, skills, and abilities. Diverse groups are known to be more creative in their decision processes as a result of their diversity and tend to attain more creative solutions to issues being addressed [59,66]. Therefore, advocates of various positions and backgrounds can be appointed in order to ensure a multitude of perspectives are brought into the policy-making decision process. This will also help to balance out the challenge of overcoming the influence of individual affect. Decision processes involving numerous people are more likely to strike a balance among affect states, thereby minimizing a dominant affect influence and balance risk taking.

5. The final strategy to counter factors that impede optimal policy decision making, such as satisficing and heuristic use, links back to point two (translating research findings into evidence that is amenable to the end-users) and the way in which research (evidence) is compiled for end-users. To utilize evidence and minimize cognitive shortcutting, the following steps will be useful:

a. As noted, health services research, aggregated across studies and translated into reliable and valid findings, is a key to evidence-based decisions. This information needs to be readily available to decision makers in the policy formulation process. Availability of translatable data would expand the individual experience factor and become part of the information basis that influence decision making.

b. The three heuristics discussed were availability, representativeness, and anchoring and adjustment. Policy research papers and briefs should recognize these heuristics and focus on summaries that increase availability of relevant information, articulate data that clarifies best practice of similar problems and issues, and provide data on relevant anchors, baseline, and tracked performance indicators such as the scorecards used by many agencies and organizations.

c. Finally, organizational commitment to educating and training key decision makers in decision-making processes will help provide the foundation and knowledge to assist individuals in recognizing when heuristics are being used and providing the opportunity for intervention if the heuristics are detrimental to the policy decision. Training key individuals in decision-making skills is as valuable to policy making as teaching negotiation skills is to those who participate in workplace negotiation, union contracts, and conflict resolution.

All of the above suggestions were made to encourage and support the discussion of alternatives to improve the health policy decision process and, ultimately, the delivery of health services across the globe. Increased recognition of the inherent biases and individual decision-making flaws is a first step of aligning policy goals with decision utilities. Additional alignment may be achieved by dedicated efforts to improve the cognitive information process and the information available to policy makers.

In presenting our cognitive information-processing framework, we contribute to the health policy decision literature by developing a framework that captures a wide variety of factors influencing decision-making situations. Furthermore, we argue that these considerations are globally relevant and that a comprehensive understanding of the mechanisms of cognitive processing aids decision makers in developing awareness of how they process relevant decision information and how they may be subtly influenced while discounting actual evidence. In addition, empirical studies could be designed to test the degree to which these issues impact health policy decision in various settings. Identifying and better understanding these influences will empower both health policy makers and managers to enhance their decision-making. The mechanics of decision making and how individual cognitively process information when evaluating decision alternatives must become explicit knowledge that is utilized to aid the EBDM goals of policy makers. We posit that a greater awareness of the reasons behind policy makers' actions will promote better and more informed decisions.

## Competing interests

The authors declare that they have no competing interests.

## Authors' contributions

DM conceived and drafted the original manuscript. Both authors (DM and NSB) contributed in further drafting of the manuscript for publication, and both authors have participated in revisions for intellectual content. DM was responsible for all formatting, literature updating, responding to reviewers, and the submission process. DM and NSB have given final approval of the version of the manuscript to be submitted.

## References

[B1] USA National Health Expenditure Projections 2007-2016 http://www.cms.hhs.gov/NationalHealthExpendData/25_NHE_Fact_Sheet.asp#TopOfPage

[B2] Redd SB The Influence of Advisors on Foreign Policy Decision Making Journal of Conflict Resolution 2002 46 335 364 10.1177/0022002702046003002

[B3] Atkins D Siegel J Slutsky J Making Policy When the Evidence is in Dispute Health Affairs 2005 24 102 113 10.1377/hlthaff.24.1.102 15647220

[B4] Clancy CM Cronin K Evidence-Based Decision Making: Global Evidence, Local Decisions Health Affairs 2005 24 151 162 10.1377/hlthaff.24.1.151 15647226

[B5] Fox DM Evidence of Evidence-Based Health Policy: The Politics of Systematic Reviews in Coverage Decisions Health Affairs 2005 24 114 122 10.1377/hlthaff.24.1.114 15647221

[B6] Isen AM Lewis M, Haviland-Jones JM Positive Affect and Decision Making Handbook of Emotions 2000 2 New York: The Guilford Press 417 435

[B7] Kuvaas B Selart M Effects of Attribute Framing on Cognitive Processing and Evaluation Organizational Behavior and Human Decision Processes 2004 95 198 207 10.1016/j.obhdp.2004.08.001

[B8] Simon AF Fagley NS Halleran JG Decision Framing: Moderating Effects of Differences and Cognitive Processing Journal of Behavioral Decision Making 2004 17 77 93 10.1002/bdm.463

[B9] Wyer RS Srull TK Hastie R, Ostrom TM, Ebbesen EB, Wyer RS, Hamilton DL, Carlston DE The Processing of Social Stimulus Information: A Conceptual Integration Person Memory: The Cognitive Basis of Social Perception 1980 Hillsdale: Lawrence Erlbaum Associates 227 300

[B10] Wyer RS Srull TK Human Cognition in its Social Context Psychological Review 1986 93 322 359 10.1037/0033-295X.93.3.322 3749400

[B11] Flemming K Fenton M Thompson C, Dowding D Making Sense of Research Evidence to Inform Decision-Making Clinical Decision-Making and Judgment in Nursing 2002 Toronto: Harcourt Publishers 110 129

[B12] Murnighan JK Mowen JC The Art of High-Stakes Decision Making 2002 New York: John Wiley and Sons

[B13] Nutley SM Walter I Davies HTO Using Evidence: How Research Can Inform Public Services 2007 The Policy Press

[B14] Sanderson I Making Sense of 'What Works': Evidence Based Policy Making as Instrumental Rationality? Public Policy and Administration 2002 17 61 75 10.1177/095207670201700305

[B15] Soumerai SB Ross-Degnan D Fortress EE Walser BL Determinants of Change in Medicaid Pharmaceutical Cost Sharing: Does Evidence Affect Policy? The Milbank Quarterly 1997 75 11 34 10.1111/1468-0009.00043 9063299 PMC2751039

[B16] Decter MB Four Strong Winds: Understanding the Growing Challenges to Health Care 2000 Toronto: Stoddart

[B17] Goldsteen RL Goldsteen K Swan JH Clemena W Harry and Louise and Health Care Reform: Romancing Public Opinion Journal of Health Politics, Policy, and Law 2001 26 1325 1352 10.1215/03616878-26-6-1325 11831582

[B18] Rodwin MA The Politics of Evidence-Based Medicine Journal of Health Politics, Policy, and Law 2001 26 439 446 10.1215/03616878-26-2-439 11330089

[B19] Howlett M Ramesh M Studying Public Policy: Policy Choices and Policy Subsystems 2003 Don Mills: Oxford University Press

[B20] Lomas J Fulop N Gagnon D Allen P On Being a Good Listener: Setting priorities for Applied Health Services Research The Milbank Quarterly 2003 81 363 388 10.1111/1468-0009.t01-1-00060 12941000 PMC2690239

[B21] Manfredi CP Maioni A Courts and Health Policy: Judicial Policy Making and Publicly Funded Health Care in Canada Journal of Health Politics, Policy, and Law 2001 27 213 240 10.1215/03616878-27-2-213 12043895

[B22] Lavis JN Ross SE Hurley JE Stoddart GL Woodward C Abelson J Giacomini M Examining the Role of Health Services Research in Public Policymaking The Milbank Quarterly 2002 80 125 154 10.1111/1468-0009.00005 11933791 PMC2690103

[B23] Lavis JN Robertson D Woodside JM McLeod CB Abelson J Knowledge Transfer Study Group How Can Research Organizations More Effectively Transfer Research Knowledge to Decision Makers? The Milbank Quarterly 2003 81 221 245 10.1111/1468-0009.t01-1-00052 12841049 PMC2690219

[B24] Biller-Adorno N Lie RK Meulen RT Evidence-Based Medicine as an Instrument for Rational Health Policy Health Care Analysis 2002 10 261 275 10.1023/A:1022947707243 12769414

[B25] Lin V Gibson B Evidence-Based Health Policy 2003 Melbourne: Oxford University Press

[B26] Black N Evidence Based Policy: Proceed With Care British Medical Journal 2001 323 275 279 10.1136/bmj.323.7307.275 11485961 PMC1120888

[B27] Sanderson I Evaluation, Policy Learning and Evidence-Based Policy Making Public Administration 2002 80 1 22 10.1111/1467-9299.00292

[B28] Tversky A Kahneman D The Framing of Decisions and the Psychology of Choice Science 1981 211 453 458 10.1126/science.7455683 7455683

[B29] Weiss C Bucuvalas MJ Social Science Research and Decision Making 1980 New York: Columbia University Press

[B30] Jones BD Politics and the Architecture of Choice: Bounded Rationality and Governance 2001 Chicago: University of Chicago Press

[B31] Sabatier PA Sabatier PA Fostering the Development of Policy Theory Theories of the Policy Process 1999 Boulder: Westview Press 3 17

[B32] Simon HA Zey M Decision Making and Problem Solving Decision Making: Alternatives to Rational Choice 1992 Newbury Park: Sage Publications 32 53

[B33] Mulrow CD Lohr KN Proof and Policy From Medical Research Evidence Journal of Health Politics, Policy, and Law 2001 26 249 266 10.1215/03616878-26-2-249 11330080

[B34] Peterson M Editor's Note - Evidence: Its Meanings in Health Care and Law Journal of Health Politics, Policy, and Law 2001 26 191 193 10.1215/03616878-26-2-191 11441806

[B35] Greenhalgh T Robert G Macfarlane F Bate P Kyriakidou O Diffusion of Innovations in Service Organizations: Systematic Review and Recommendations The Milbank Quarterly 2004 82 581 629 10.1111/j.0887-378X.2004.00325.x 15595944 PMC2690184

[B36] Vos R Houtepen R Horstman K Evidence-Based Medicine and Power Shifts in Health Care Systems Health Care Analysis 2002 10 319 328 10.1023/A:1022908025898 12769419

[B37] Dobrow MJ Goel V Upshur REG Evidence-Based Health Policy: Context and Utilization Social Sciences and Medicine 2004 58 207 217 10.1016/S0277-9536(03)00166-7 14572932

[B38] Jacobson N Butterill D Goering P Consulting as a Strategy for Knowledge Transfer The Milbank Quarterly 2005 83 299 321 10.1111/j.1468-0009.2005.00348.x 15960773 PMC2690143

[B39] Gold M The Changing US Health Care System: Challenges for Responsible Public Policy The Milbank Quarterly 1999 77 3 37 10.1111/1468-0009.00123 10197026 PMC2751111

[B40] Walshe K Rundall TG Evidence-Based Management: From Theory to Practice in Health Care The Milbank Quarterly 2001 79 429 457 10.1111/1468-0009.00214 11565163 PMC2751196

[B41] Lin V Lin V, Gibson B Competing Rationalities: Evidence-Based Health Policy? Evidence-Based Health Policy 2003 Melbourne: Oxford University Press 3 17

[B42] Willis E White K Lin V, Gibson, B Evidence-Based Medicine, the Medical Profession, and Health Policy Evidence-Based Health Policy 2003 Melbourne: Oxford University Press 33 55

[B43] Zarkovich E Upshur REG The Virtues of Evidence Theoretical Medicine 2002 23 403 412 10.1023/A:1021217908383 12516841

[B44] Simon HA A Behavioral Model of Rational Choice Quarterly Journal of Economics 1955 69 99 118 10.2307/1884852

[B45] Maynard RA Presidential Address - Evidence-Based Decision Making: What Will it Take for the Decision Makers to Care? Journal of Policy Analysis and Management 2006 25 249 265 10.1002/pam.20169

[B46] Simon HA Bounded Rationality and Organizational Learning Organization Science 1991 2 125 135 10.1287/orsc.2.1.125

[B47] Kahneman D Tversky A Prospect Theory: An Analysis of Decision Under Risk Econometrica 1979 47 263 291 10.2307/1914185

[B48] Zey M Zey M Criticisms of Rational Choice Models Decision Making: Alternatives to Rational Choice Models 1992 Newbury Park: Sage Publications 10 31

[B49] Fernandes R Simon HA A Study of How Individuals Solve Complex and Ill-Structured Problems Policy Sciences 1999 32 225 245 10.1023/A:1004668303848

[B50] Agosto DE Bounded Rationality and Satisficing in Young Peoples' Web-Based Decision Making Journal of the American Society for Information Science and Technology 2001 53 16 27 10.1002/asi.10024

[B51] Gupta M A Critical Appraisal of Evidence-Based Medicine: Some Ethical Considerations Journal of Evaluation in Clinical Practice 2003 9 111 121 10.1046/j.1365-2753.2003.00382.x 12787171

[B52] Norman G Examining the Assumptions of Evidence-Based Medicine Journal of Evaluation in Clinical Practice 1999 5 139 147 10.1046/j.1365-2753.1999.00197.x 10471222

[B53] Estabrooks CA Morse JM, Swanson J, Kuzel AF Research Utilization and Qualitative Research The Nature of Qualitative Evidence 2001 Thousand Oaks: Sage Publications 275 298

[B54] Jones BD Reconceiving Decision-Making in Democratic Politics: Attention, Choice, and Public Policy 1994 Chicago: University of Chicago Press

[B55] Haddow R Bickerton J, Gagnon A Interest Representation and the Canadian State Canadian Politics 1999 Peterborough: Broadview Press 501 522

[B56] Brooks S Miljan L Public Policy in Canada: An Introduction 2003 Toronto: Oxford University Press

[B57] Stone D Capturing the Political Imagination: Think Tanks and the Policy Process 1996 Portland: Frank Cass

[B58] Lightle JP Kagel JH Arkes HR Information Exchange in Group Decision Making: The Hidden Profile Problem Reconsidered Management Science 2009 55 568 581 10.1287/mnsc.1080.0975

[B59] Allison G Zelikow P Essence of Decision: Explaining the Cuban Missile Crisis 1999 New York: Addison-WesleyEducational Publishers Inc

[B60] Zey M Zey M Criticisms of Rational Choice Models Decision Making: Alternatives to Rational Choice Models 1992 Newbury Park: Sage Publications 9 31

[B61] Spitz B Abramson J When Health Policy is the Problem: A Report From the Field Journal of Health Politics, Policy, and Law 2005 30 327 365 10.1215/03616878-30-3-327 16089109

[B62] Johnson RB Toward a Theoretical Model of Evaluation Utilization Evaluation and Program Planning 1998 21 93 110 10.1016/S0149-7189(97)00048-7

[B63] Srull TK Wyer RS Sorrentino RM, Higgins ET The Role of Chronic and Temporary Goals in Social Information Processing Handbook of Motivation and Cognition: Foundations of Social Behavior 1986 New York: The Guilford Press 503 549

[B64] Lord RG Maher KJ Cooper CL, Robertson IT Cognitive Processes in Industrial and Organizational Psychology International Review of Industrial and Organizational Psychology 1989 New York: John Wiley and Sons 49 91

[B65] Lord RG Maher KJ Leadership and Information Processing 1991 Cambridge: Unwin Hyman Inc

[B66] Bazerman MH Judgment in Managerial Decision Making 1998 4 New York: John Wiley and Sons

[B67] Slovic P Monahan J Slovic, P Probability, Danger, and Coercion: A Study of Risk Perception and Decision Making in Mental Health Law The Perception of Risk 2000 Sterling: Earthscan Publications Ltd 347 36

[B68] Williams S Zainuba M Jackson R Affective Influences on Risk Perceptions and Risk Intentions Journal of Managerial Psychology 2003 18 126 137 10.1108/02683940310465027

[B69] Brief AP Weiss HW Organizational Behavior: Affect in the Workplace Annual Review of Psychology 2002 53 279 307 10.1146/annurev.psych.53.100901.135156 11752487

[B70] Ubel PA Baron J Asch DA Preference for Equity as a Framing Effect Medical Decision Making 2002 21 180 189 10.1177/0272989X0102100303 11386625

[B71] Levin IP Schneider SL Gaeth GJ All Frames are not Created Equal: A Typology and Critical Analysis of Framing Effects Organizational Behavior and Human Decision Processes 1998 76 149 188 10.1006/obhd.1998.2804 9831520

[B72] McElroy T Seta JJ Framing Effects: An Analytic-Holistic Perspective Journal of Experimental Social Psychology 2003 39 610 617 10.1016/S0022-1031(03)00036-2

[B73] Mittal V Ross WT The Impact of Positive and Negative Affect and Issue Framing on Issue Interpretation and Risk Taking Organizational Behavior and Human Decision Processes 1998 76 298 324 10.1006/obhd.1998.2808 9878512

[B74] Bless H Betsch T Franzen A Framing the Framing Effect: The Impact of Context Cues on Solutions to the 'Asian Disease' Problem European Journal of Social Psychology 1999 28 287 291 10.1002/(SICI)1099-0992(199803/04)28:2<287::AID-EJSP861>3.0.CO;2-U

[B75] Slovic P Kunreuther H White G Slovic, P Decision Processes, Rationality, and Adjustment to Natural Hazards The Perception of Risk 2000 Sterling: Earthscan Publications Ltd 1 31

[B76] Eich E Schooler JW Eich E, Kihlstrom JF, Bower GH, Forgas JP, Niedenthal PM Cognition/Emotion Interactions Cognition and Emotion 2000 New York: Oxford University Press 87 168

[B77] Forgas JP Vargas PT Lewis M, Haviland-Jones JM The Effects of Mood on Social Judgment and Reasoning Handbook of Emotions 2000 2 New York: The Guilford Press 350 367

[B78] Johnson-Laird PN Oatley K Lewis M, Haviland-Jones JM Cognitive and Social Construction in Emotions Handbook of Emotions 2000 2 New York: The Guilford Press 458 475

[B79] Bower GH Forgas JP Forgas JP Mood and Social Memory Handbook of Affect and Social Cognition 2001 Mahwah: Lawrence Erlbaum Associates, Inc 95 120

[B80] Clore GL Gasper K Garvin E Forgas JP Affect as Information Handbook of Affect and Social Cognition 2001 Mahwah: Lawrence Erlbaum Associates, Inc 121 144

[B81] George JM State or Trait: Effects of Positive Mood on Prosocial Behaviors at Work Journal of Applied Psychology 1989 76 299 307 10.1037/0021-9010.76.2.299

[B82] George JM Mood and Absence Journal of Applied Psychology 1991 74 317 324 10.1037/0021-9010.74.2.317

[B83] Wright TA Cropanzano R Meyer DG State and Trait Correlates of Job Performance: A Tale of Two Perspectives Journal of Business and Psychology 2004 18 365 383 10.1023/B:JOBU.0000016708.22925.72

[B84] Czajka J The Relation of Positive and Negative Affectivity to Workplace Attitudes Academy of Management Proceedings 1990 201 205

[B85] Cropanzano R James K Konovsky MA Dispositional Affectivity as a Predictor of Work Attitudes and Job Performance Journal of Organizational Behavior 1993 14 595 606 10.1002/job.4030140609

[B86] Adolphs R Damasio AR Forgas JP The Interaction of Affect and Cognition: A Neurobiological Perspective Handbook of Affect and Social Cognition 2001 Mahwah: Lawrence Erlbaum Associates, Inc 27 49

[B87] Daniels K Towards Integrating Emotions into Strategic Management Research: Trait Affect and Perceptions of the Strategic Environment British Journal of Management 1998 9 163 168 10.1111/1467-8551.00081

[B88] Forgas JP Forgas JP Affect and Information Processing Strategies: An Interactive Relationship Feeling and Thinking: The Role of Affect in Social Cognition 2000 New York: Cambridge University Press 253 282

[B89] Forgas JP Mood and Judgment: The Affect Infusion Model (AIM) Psychological Bulletin 1995 117 39 66 10.1037/0033-2909.117.1.39 7870863

[B90] Forgas JP Forgas JP Affect, Cognition, and Interpersonal Behavior: The Mediating Role of Processing Strategies Handbook of Affect and Social Cognition 2001 Mahwah: Lawrence Erlbaum Associates, Inc 293 318

[B91] Jundt DK Hinsz VB Influences of Positive and Negative Affect on Decisions Involving Judgmental Biases Social Behavior and Personality 2002 30 45 52 10.2224/sbp.2002.30.1.45

[B92] Fiedler K Forgas JP Affective Influences on Social Information Processing Handbook of Affect and Social Cognition 2001 Mahwah: Lawrence Erlbaum Associates, Inc 163 185

[B93] Forgas JP George JM Affective Influences on Judgments and Behavior in Organizations: An Information Perspective Organizational Behavior and Human Decision Processes 2001 86 3 34 10.1006/obhd.2001.2971

[B94] Slattery JP Ganster DC Determinants of Risk Taking in a Dynamic Uncertain Context Journal of Management 2002 28 89 106 10.1177/014920630202800106

[B95] Slovic P Slovic P Perception of Risk The Perception of Risk 2000 Sterling: Earthscan Publications Ltd 220 231

[B96] Vaccines & Preventable Diseases http://www.cdc.gov/vaccines/vpd-vac/default.htm

[B97] Kuhn TS The Structure of Scientific Revolutions 1996 3 Chicago: The University of Chicago Press

[B98] Kuhn TS Kourany JA The Nature and Necessity of Scientific Revolutions Scientific Knowledge: Basic Issues in the Philosophy of Science 1998 2 Belmont: Wadsworth Publishing Company 316 326

[B99] Kourany JA Kourany JA The Validation of Scientific Knowledge Scientific Knowledge: Basic Issues in the Philosophy of Science 1998 2 Belmont: Wadsworth Publishing Company 153 163

[B100] Sackett DL Rosenberg WMC Gray JAM Haynes RB Richardson WS Evidence-Based Medicine: What it is and What it isn't British Medical Journal 1996 312 71 72 8555924 10.1136/bmj.312.7023.71 PMC2349778

[B101] Burrell G Morgan G Sociological Paradigms and Organizational Analysis 1979 London: Heinemann Books

[B102] Innvaer S Vist G Trommald M Oxman A Health Policy-Makers' Perceptions of Their Use of Evidence: A Systematic Review Journal of Health Services Research and Policy 2002 7 239 244 10.1258/135581902320432778 12425783

[B103] Garvin DA Roberto MA What You Don't Know About Making Decisions Harvard Business Review 2001 108 116 11550627

[B104] Delbecq AL Van de Ven AH A Group Process Model for Problem Identification and Program Planning Journal of Applied Behavioral Science 1971 7 466 491 10.1177/002188637100700404

